# Study on the Molecular Mechanism of the Herbal Couple Sparganii Rhizoma-Curcumae Rhizoma in the Treatment of Lung Cancer Based on Network Pharmacology

**DOI:** 10.1155/2021/6664489

**Published:** 2021-06-18

**Authors:** Shuying Dai, Gaochenxi Zhang, Fangmin Zhao, Qijin Shu

**Affiliations:** ^1^Department of First Clinical Medical College, Zhejiang Chinese Medical University, Hangzhou 310053, China; ^2^Department of Oncology, Zhejiang Provincial Hospital of Traditional Chinese Medicine, Hangzhou 310006, China

## Abstract

**Background:**

Lung cancer has a poor prognosis and a high mortality rate, and patients may develop multidrug resistance. Sparganii Rhizoma-Curcumae Rhizoma (HCSC), the classic herbal drug combination of traditional Chinese medicine (TCM), is commonly used in treating tumors, but its molecular mechanism is still unclear.

**Method:**

We explored the possible mechanisms underlying the antitumor effect of HCSC using network pharmacology. The bioactive components of HCSC and their targets were collected from the TCM Systems Pharmacology (TCMSP) database and PharmMapper. Gene Ontology (GO) and KEGG enrichment analyses were performed; the GeneMANIA platform was used for the functional enrichment analysis of the core targets and their neighboring genes. Molecular docking was performed between the bioactive components and core targets. HCSC freeze-dried powder was prepared, and the bioactive components were verified by liquid chromatography- (LC-) mass spectrometry (MS). Human lung adenocarcinoma H1975 cells were cultured to verify *in vitro* the molecular mechanism of action of HCSC in treating lung cancer, as predicted by network pharmacology. Finally, we used the Symmap database to predict the relationship between the herb and TCM syndrome.

**Result:**

A total of seven bioactive components were identified by network pharmacological analysis. Through enrichment analyses, it was found that the mechanism of action mainly involved mitochondrial-mediated caspase-dependent cell apoptosis signaling pathways. The results of molecular docking showed that the bioactive components in HCSC have a good affinity with the target proteins (ALB, BCL2L1, ESR1, HRAS, MAP2K1, MAPK14, and SIRT1). LC-MS confirmed that formononetin and bisdemethoxycurcumin were present in the HCSC freeze-dried powder, consistent with the prediction. The results of *in vitro* experiments on NCI-H1975 cells confirmed that HCSC can upregulate the mitochondrial-mediated caspase-dependent apoptosis signaling pathway by inducing the cleavage of caspase-3, caspase-9, and PARP, consistent with the network pharmacology prediction. Further, the qi deficiency and blood stasis associated with TCM syndrome can be treated with HCSC.

## 1. Introduction

Lung cancer is one of the most common malignancies worldwide; it is associated with high mortality rates, and its incidence has been increasing gradually in recent years [1]. Histopathologically, lung cancer is primarily divided into small-cell lung cancer (SCLC, approximately 15% of all cases) and non-small-cell lung cancer (NSCLC, approximately 85% of all cases). The main treatment modalities include surgery, radiotherapy, chemotherapy, and molecular-targeted therapy; however, these modalities are expensive and have poor long-term efficacy and obvious side-effects [2]. The 5-year survival rate of SCLC is only 19%, whereas the 5-year survival rate of NSCLC is even less, at 10%. At lung cancer diagnosis, approximately 75% of all patients are in the advanced stage (stage III/IV) and cannot undergo radical surgery [3, 4]. The occurrence and development of lung cancer are complex processs, and single-target treatments are often unable to achieve the best curative effect. Traditional Chinese medicine (TCM) includes various bioactive components that act on multiple targets and pathways in lung cancer treatment; this approach is consistent with the idea of multitarget treatment for cancer.

There has been no clear name for “lung cancer” in the history of TCM. Currently, it is mostly classified into the categories “feiji,” “xiben,” “lung distension,” or “hemoptysis.” Shen Jinao, a medical expert of the Qing Dynasty, wrote a book called “Incisive Light on the Source of Miscellaneous Disease,” which pointed out that “evil qi accumulates in the chest, blocks the airway, inhibits the functioning of qi, which causes phlegm-rheum, food accumulation, blood stasis, and the vacuity of right qi. Then it clumped.” Based on the basic theory of TCM and clinical experience, it has been believed that spleen qi is damaged after treatment. Radiotherapy, chemotherapy, and molecular-targeted drugs are heat toxins as per TCM and are more likely to wear qi and damage yin. Therefore, the deficiency of qi and yin was always reported throughout the entire process of lung cancer. This habitus makes patients more prone to qi stagnation and blood stasis.

Sparganii Rhizoma is the dried rhizome of *Sparganium stoloniferum*. Curcumae Rhizoma, also known as turmeric, is a dried rhizome of common turmeric. According to TCM, the herbal couple Sparganii Rhizoma-Curcumae Rhizoma (HCSC) activates blood circulation to dissipate blood stasis. It is mentioned in “Records of Chinese Medicine with Reference to Western Medicine” that Zhang Xichun often used HCSC in clinical practice and called it “the most important medicine for blood quickening.” It was used to treat patients with lung cancer whose right qi could still fight against evil qi to prevent metastasis and recurrence after treatment. HCSC has been clinically proven to relieve the symptoms of patients with tumors, especially gynecological ones [5]. Modern pharmacology has proven that nonvolatile fractions of HCSC possess extensive anticancer, anti-inflammatory, and antithrombotic activities [6–8]. The antitumor effect of HCSC is synergistic [7]. Curcumin can induce tumor cell apoptosis by upregulating the expression of Caspase-9, Caspase-3, Caspase-7, and poly(adenosine diphosphate-ribose) polymerase (PARP), and it displays antineoplastic effects in tumor-bearing mice [9–11].

**Figure 1:**
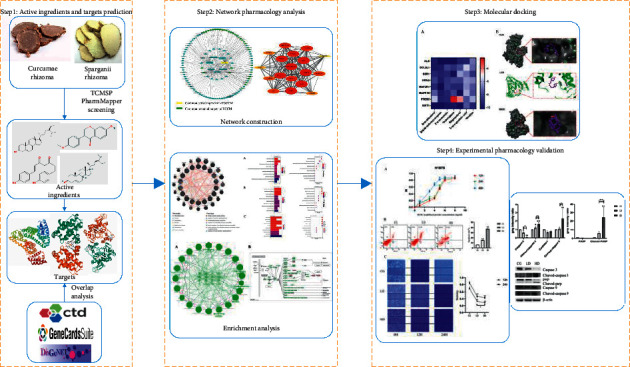
Schematic diagram for identifying the mechanism of HCSC antilung cancer by network pharmacology analysis.

Network pharmacology is a discipline that combines systems biology and multidirectional pharmacology based on high-throughput omics data analysis, virtual computer calculation, and network database retrieval [12]. Network pharmacology has been used to study multiple protein/gene target diseases and can describe the relationship between biological systems, drugs, and diseases from the perspective of the network, which is consistent with the holistic pattern differentiation theory of TCM [13]. As a multicomponent medicine, the network pharmacology method can be used to visualise the complex mechanisms of TCM. In this study, the bioactive components of HCSC were analysed and the relevant targets and signaling pathways in the treatment of lung cancer were studied in detail. In particular, NSCLC has a poor prognosis and a high mortality rate, and most patients seeking TCM treatment in clinical practice have developed multidrug resistance. The T790M mutation is one of the main mechanisms of acquired drug resistance in the first generation of epidermal growth factor receptor tyrosine kinase inhibitor- (EGFR-TKI-) targeted drugs. Therefore, we used human lung adenocarcinoma H1975 cells to verify the molecular mechanism of action of HCSC in the treatment of lung cancer, as predicted by network pharmacology through *in vitro* experiments. The detailed flowchart of the current study was shown in Figure 1.

## 2. Materials and Methods

### 2.1. Identification of Bioactive Components and Targets of HCSC

The bioactive components of HCSC were retrieved from the TCM Systems Pharmacology (TCMSP) platform (http://lsp.nwu.edu.cn/tcmsp.php) for Chinese herbal medicines; this platform captures the relationships between drugs, targets, and diseases [14]. The screening conditions were oral bioavailability (OB) of >30% and drug-likeness (DL) of >0.18. The reverse docking server PharmMapper (http://lilab.ecust.edu.cn/pharmmapper/) was used to simulate the molecular docking of the bioactive components [15]. The range was set to “Human Protein Targets Only (v2010, 2241)”, and the other parameters were set at default. The screening criterion was a fit score, which could reflect the combination of ligand configuration and the pharmacophore model. Ten targets with the highest value were selected. Drug targets collected through TCMSP and PharmMapper were integrated. Related gene information, including gene name, gene ID, and gene symbol, belonging to “*Homo sapiens*,” was collected from the UniProt database (https://www.uniprot.org/) [16].

### 2.2. Prediction of Potential Targets in Lung Cancer

The Comparative Toxicogenomics Database (http://ctdbase.org/) [17], GeneCards (https://auth.lifemapsc.com/) [18], and DisGeNET (https://www.disgenet.org/) [19] were searched using keywords such as “lung cancer” and “lung neoplasms” to obtain disease targets. The overlapping part after integrating the disease targets and drug targets was considered the candidate target of HCSC against lung cancer.

### 2.3. Network Construction

A protein-protein interaction (PPI) network of candidate targets was constructed using the STRING database (https://string-db.org/) [20]; the “minimum required interaction score” was adjusted to ensure readability. The network data were then exported. Next, the targets in the network data were integrated and deweighted to obtain potential targets. Drugs, bioactive components, and corresponding potential targets were imported into Cytoscape (version 3.7.1) [21] to construct a “drugs-bioactive components-potential targets” network. In addition, node1, node2, and combined scores were imported into the network data into Cytoscape. The plugin MCODE in Cytoscape was used to perform cluster analysis of the potential targets. The targets in cluster 1 were screened as core targets, and a PPI network was constructed.

### 2.4. Enrichment Analysis

GeneMANIA (http://www.genemania.org/) [22] was used to construct a network of core targets and neighboring genes while performing functional enrichment analysis. RGUI and clusterProfiler [23] were used to perform the GO enrichment analysis of core targets to obtain the biological processes, cellular components, and molecular function (*P* < 0.05). The ClueGO and CluePedia plugins in Cytoscape were used to perform the KEGG enrichment analysis of the core targets (*P* < 0.05); then, the pathway nodes in the enrichment network were manually selected. Candidate targets were converted to UniProtID using the UniProt software. The “search&Color Pathway” tool of the KEGG database (https://www.kegg.jp/kegg/pathway.html) [24] was used to map the candidate targets to the related pathways.

### 2.5. Molecular Docking Verification

The HCSC bioactive components (ligands) were processed using ChemBio 3D software and stored in the mol2 format. Then, AutoDock Tools [25] were used to save it in PDBQT format for future use; the proteins were preprocessed, excess protein chains and ligands were removed, and water molecules were hydrogenated. AutoGrid was used for energy grid calculation. Next, we determined the PDB-ID and downloaded the crystal structure of the target proteins containing the original ligand in the PDB database (http://www.rcsb.org/) [26]. The original ligand was extracted from the target proteins using DiscoveryStudio2016 software and then redocked to the active site of the complex using AutoDock software. The root mean square deviation (RMSD) between the result conformation and the target conformation was compared; if the RMSD obtained was >2 Å, the protein docking effect was deemed not good and the corresponding target protein was discarded. AutoDock Vina was used to conduct molecular docking of the ligand and core targets. After the docking was completed, △G was used as a reference to screen the target of the active compound. If △G was <0, it was considered that the ligand and target protein can bind spontaneously. △*G* ≤ −5.0 kJ mol^−1^ was selected as the screening basis. The dominant conformation was analysed and plotted using Schrodinger.

### 2.6. *In Vitro* Experimental Verification in Cells

#### 2.6.1. Preparation of HCSC Aqueous Extract

HCSC consisted of 45 g of Sparganii Rhizoma and Curcumae Rhizoma each. All crude herbs were provided by the Zhejiang Provincial Hospital of Traditional Chinese Medicine. The herbs were mixed and boiled thrice in 1000 mL double-steamed water for 30 min each time, filtered by vacuum filtration, and concentrated on an induction cooker to about 100 mL. The liquid was then evaporated to dryness in a vacuum at −40°C. The final freeze-dried powder weighed 2.93 g; the yield was 3.25% (calculated at 100% concentration). The freeze-dried powder of HCSC was then dissolved in double-steamed water (10 mg/mL) and filtered through a microporous filter for sterilisation.

#### 2.6.2. Cell Culture

Human lung adenocarcinoma H1975 cells (EGFR exon 20 T790M-L858R, gefitinib-resistant cell line) were kindly provided by Professor Zhou Caicun (Shanghai Pulmonary Hospital affiliated with Tongji University, China). The cells were cultured in Dulbecco's modified Eagle's medium supplemented with 10% foetal bovine serum, 100 U/mL penicillin, and 100 mg/mL streptomycin and maintained in a humidified chamber with 5% CO_2_ at 37°C. Cells in the logarithmic growth phase were used for all the experiments.

#### 2.6.3. Cell Viability Assay

A total of 6 × 10^3^ logarithmically growing H1975 cells/well were seeded in 96-well plates. After incubation for 24 h until the cell monolayer covered the bottom of the well, the cells were treated with varying concentrations of HCSC (8, 6, 5, 4, 3, and 1 mg/mL) for 24, 48, and 72 h. The 10% Cell Counting Kit-8 (CCK-8; Dojindo, JAPAN) solution was added to each well, and the cells were cultured for another 1 h at 37°C. The optical density (OD) was measured at 450 nm using a multifunctional microplate reader (Thermo Fisher Scientific, USA). The inhibition rate is (IR% = (average OD value of control group - average OD average of experimental group)/average OD value of control group × 100%). The experiment was repeated three times in parallel. SPSS software was used to calculate the IC_20_ and IC_50_ values.

### 2.7. ANNEXIN V-FITC/PI Double Staining and Flow Cytometry

Cells (1 × 10^5^ cells/well) were seeded into 6-well plates. After incubation for 24 h, the cells were divided into the control (CG), low-dose (LD), and high-dose (HD) groups. The CG was treated without HCSC, while the LD and HD groups were treated with IC_20_ and IC_50_ proportions of HCSC for 24 h. The cells were collected and suspended in a 100 *μ*l binding buffer containing 5 *μ*l Annexin V-FITC and 10 *μ*l PI. After incubation in the dark for 15 min at room temperature, 400 *μ*l of binding buffer was added. Flow cytometry (BD Bioscience, USA, NO. 556547) was used to detect cell apoptosis in each group, and the FACSDiva Version 6.1.3 software was used for data analysis.

### 2.8. Wound Healing Assay

For the wound healing assay, cells were seeded in 6-well plates and incubated until 100% confluence. A sterile 200 *μ*L pipette tip was used to scrape a denuded area on the cell monolayer. After washing thrice with phosphate-buffered saline, the cells were treated with HCSC as described above. After incubation for 0, 12, and 24 h, cell migration to the wounded region was monitored using NIS-Elements F3.2 for Windows XP/Professional. To quantify cell migration, images of the initially wounded monolayers were equated to the corresponding pictures of cells at later time points. The migrated cells in each of the three random fields were counted using the following formula: Mobility (%) = (1–0 h wound area/12 or 24 h wound area) × 100%. Image J was used to analyse the image data and determine the scratch area. The experiment was repeated three times in parallel.

### 2.9. Western Blotting

Cells (1 × 10^5^ cells/well) were seeded into 6-well plates. After incubation for 24 h, the cells were treated as described above for 24 h. The cells were scraped and collected. The protein of the H1975 cells was extracted with lysis buffer, and the concentration was measured. The sample volume was adjusted according to the internal reference protein to ensure the same amount of protein in each sample. The proteins were separated using sodium dodecyl sulphate-polyacrylamide gel electrophoresis based on their different molecular masses. Then, the proteins were transferred onto polyvinylidene difluoride membranes (Immobilon-P, 0.45 mm; Millipore Co., Billerica, MA, USA) using sodium dodecyl sulphate-polyacrylamide gel electrophoresis. The membrane was then immersed in 5% skim milk diluted with tris-buffered saline with 0.1% Tween-20 for 2 h at room temperature. After that, the membrane was cut into several bands and incubated with diluted antibodies (1 : 1000) at 4°C overnight. The antibody selected the relevant proteins in the KEGG enrichment pathway. The bands were washed with tris-buffered saline with 0.1% Tween-20 before incubation with HRP-conjugated secondary antibodies (1 : 10000, Dawen Biotec, WD-0990, WD-GAR007). At room temperature, a second incubation was required for 2 h. The bands were visualised using an electrochemiluminescence system (Bio-Rad Laboratories, Inc., Hercules, CA, USA). The experiment was repeated three times in parallel.

### 2.10. Statistical Analysis

The data were analysed using SPSS 23.0, and the measurement data were expressed as *X* ± *S*. One-way analysis of variance was used for pairwise comparisons among multiple groups, and an independent sample *t*-test was used for mean comparison between two groups. Statistical significance was set at *P* < 0.05.

### 2.11. LC-MS Verification

The filtered mother liquor of the freeze-dried HCSC powder was diluted to 1 mg/mL with double-distilled water, and the sample was injected. Mass spectrometry conditions were as follows: in the MS^E^ continuum mode, the scan range was m/z100-1200. In the ESI^−^ mode, sodium formate was used to calibrate the instrument. Under the 0.1% formic acid water-acetonitrile mass spectrometry, gradient elution was performed for 75 min. LC-MS analysis was used to obtain the total ion current diagram (Waters SYNAPT G2-Si mass spectrometer, USA). The accurate molecular weight and secondary fragment information of the compound obtained from the total ion current diagram was compared with the information in the UNIFI TCM database to obtain the components.

### 2.12. Prediction of HCSC-Related Symptoms and Diseases

SymMap (https://www.symmap.org/) [27] contains comprehensive information about TCM herbs and their components, TCM symptoms, modern medicine symptoms, diseases, and the relationships between any two such entities. “Sparganii Rhizoma” and “Curcumae Rhizoma” were used as keywords in the SymMap system to predict their related TCM symptoms, TCM syndromes, modern medicine symptoms, and diseases.

## 3. Results

### 3.1. Identification of Bioactive Components and Targets of HCSC

A total of 111 bioactive components and 995 targets were identified in the TCMSP database. Eight potential bioactive components of HCSC and their corresponding targets were obtained (OB > 30%, DL > 0.18). Ten targets were selected with the highest fit score in the PharmMapper database. A total of 113 targets were integrated and deleted after duplication (Table 1). In the UniProt database, the targets were normalised to those corresponding to humans. One target could not be converted. By comparing the gene names related to lung cancer in the Comparative Toxicogenomics Database, GeneCards, and DisGeNET databases, 110 overlapping targets were reserved as candidate targets.

### 3.2. Core Target Identification for HCSC

**Figure 2:**
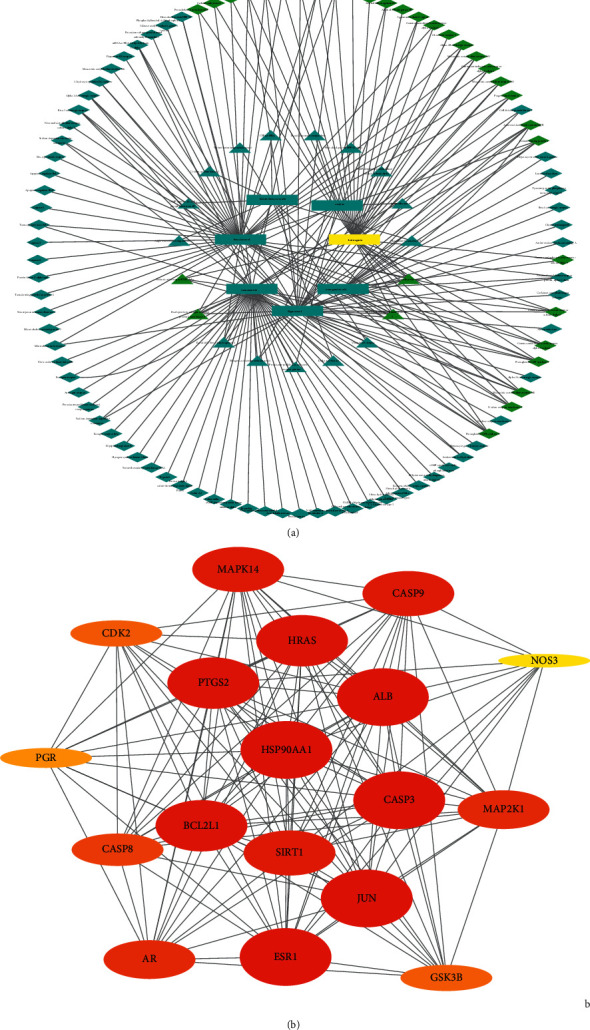
(a) “Drugs-bioactive components-potential targets” network diagram of HCSC. The square represents the bioactive components, the triangle represents the potential targets of Curcumae Rhizoma, and the diamond represents the potential targets of Sparganii Rhizoma. Lines stand for the relation of bioactive components and targets (Cytoscape). (b) PPI network diagram from core targets of HCSC. The color depth of the node and the thickness of the edge respectively represent the value of the degree and combined score (MCODE).

**Figure 3:**
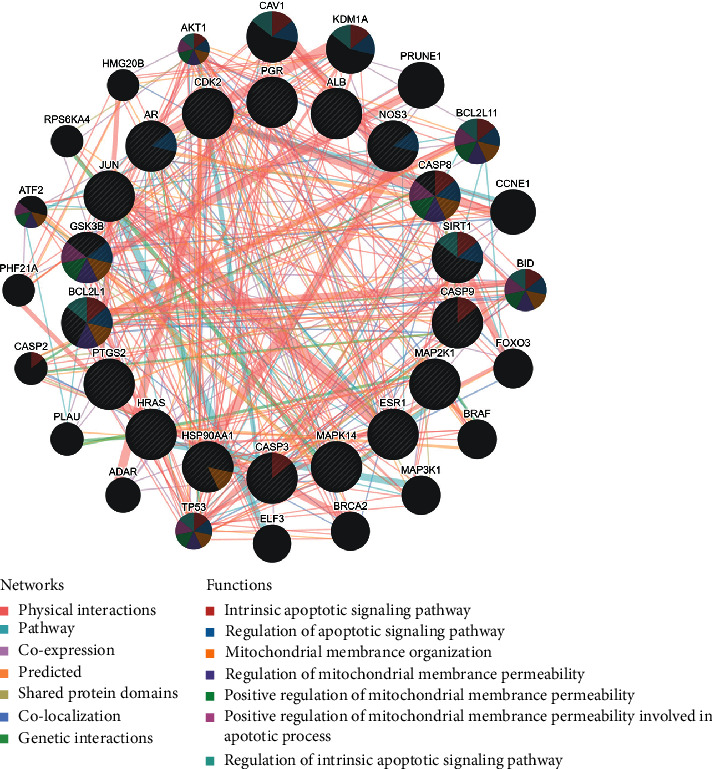
PPI network of the core targets and their neighboring genes (GeneMANIA). PPI network and functional analysis indicating the gene set that was enriched in the target network of core targets and neighboring genes. The different colors of the network edge indicate the bioinformatics methods applied: coexpression, pathway, website prediction, colocalization, shared protein domains, physical interactions, and genetic interactions. The different colors for the network nodes indicate the biological functions of the set of enrichment genes.

The above candidate targets were entered in the STRING database, and the “minimum required interaction score” was set at 0.4 to obtain a PPI network involving 106 potential targets. A “drugs-bioactive components-potential targets” network diagram was constructed using Cytoscape (Figure 2(a), 2). In addition, potential targets and combined scores in the network data were imported. According to the cluster analysis of the plugin MCODE, four subnetworks were obtained, and 18 targets in cluster1 were selected as the core targets (Figure 2(b), Table 2). The core targets were input into the GeneMANIA platform to obtain the functional enrichment analysis of the core targets and their neighboring genes (Figure 3).

### 3.3. Enrichment Analysis of the Core Targets

#### 3.3.1. GO Enrichment Analysis

**Figure 4:**
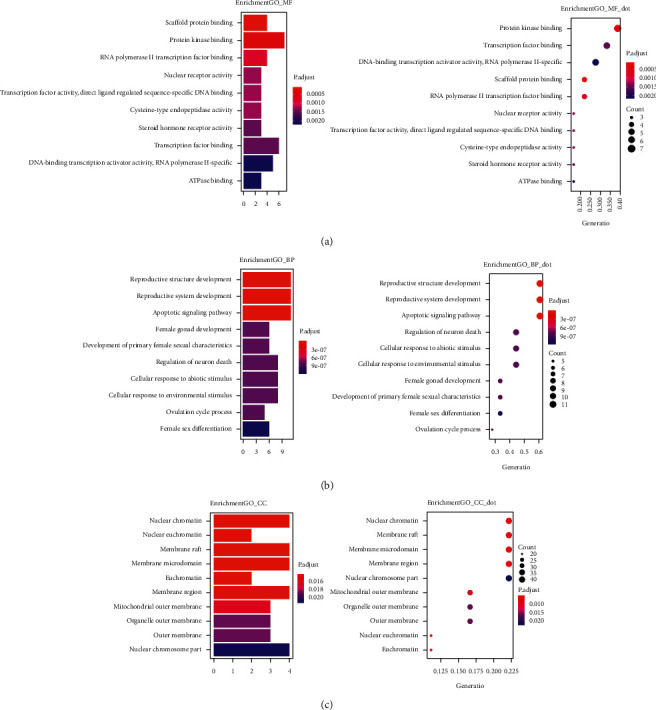
GO enrichment analysis: (a) molecular function results; (b) biological process results; (c) cellular component results.

The 18 core targets of HCSC were analysed using clusterProfiler [23] (pV < 0.05, qV < 0.05) for GO function enrichment. Bar charts and bubble charts of the GO biological processes, cellular components, and molecular function were obtained (Figure 4).

### 3.4. KEGG Enrichment Analysis

**Figure 5:**
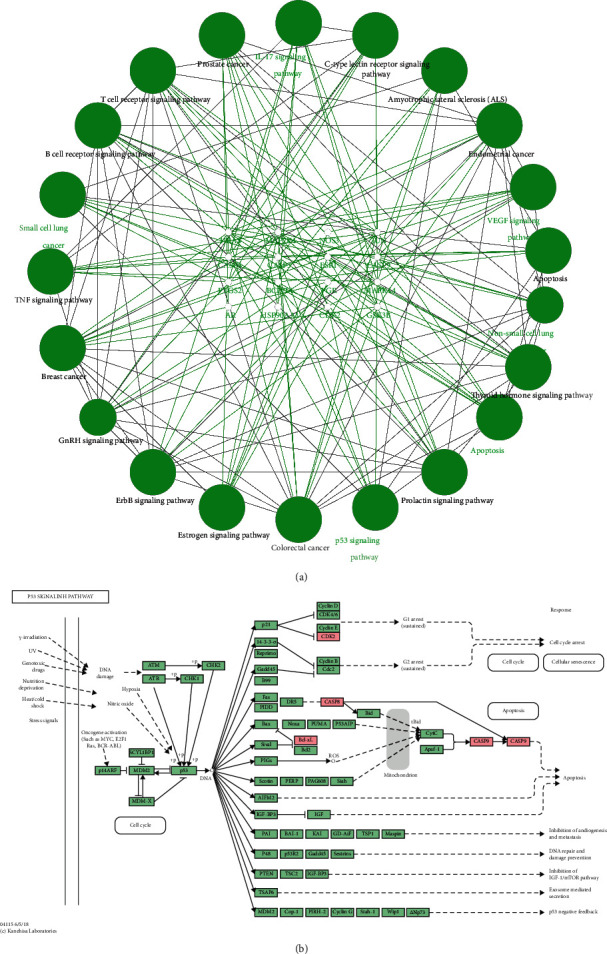
KEGG enrichment analysis. (a) Selected pathway results of the KEGG enrichment analysis; (b) P53 signaling pathway.

The core targets were analysed using ClueGO and CluePedia for the KEGG pathway enrichment analysis (*P* < 0.05; 36 items in total). In the enrichment results, the P53 signaling pathway was selected and the candidate targets were mapped. This pathway has been reported in the literature to be closely related to lung cancer [28]. The candidate targets are displayed as pink signs in the P53 signaling pathway (Figure 5).

#### 3.4.1. Molecular Docking Verification

**Figure 6:**
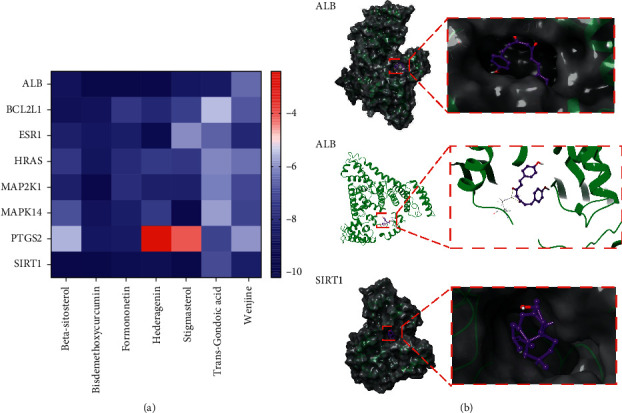
Molecular docking results. (a) Docking results of the bioactive components with target proteins; (b) dominant conformation of the bioactive components with target proteins.

AutoDock Vina was used to dock 7 bioactive components with 18 target proteins, among which 8 target proteins with RMSD values <2 Å were selected (Table 3). The results are shown in a heat map (Figure 6(a), 6). Except for the poor affinity of polygonal and stigmasterol with PTGS2, the other bioactive components have good affinity to the target proteins. Among them, sitosterol, formononetin, and curcumin III have the highest affinity to the target proteins. Stigmasterol molecules enter the active pocket of the protein and match well with the active pocket. The curcumin III molecule enters the active pocket of the ALB protein, and its phenolic hydroxyl group interacts with the ARG117 amino acid to form two hydrogen bonds (Figure 6(b)).

### 3.5. HCSC Suppresses *In Vitro* H1975 Cell Growth

**Figure 7:**
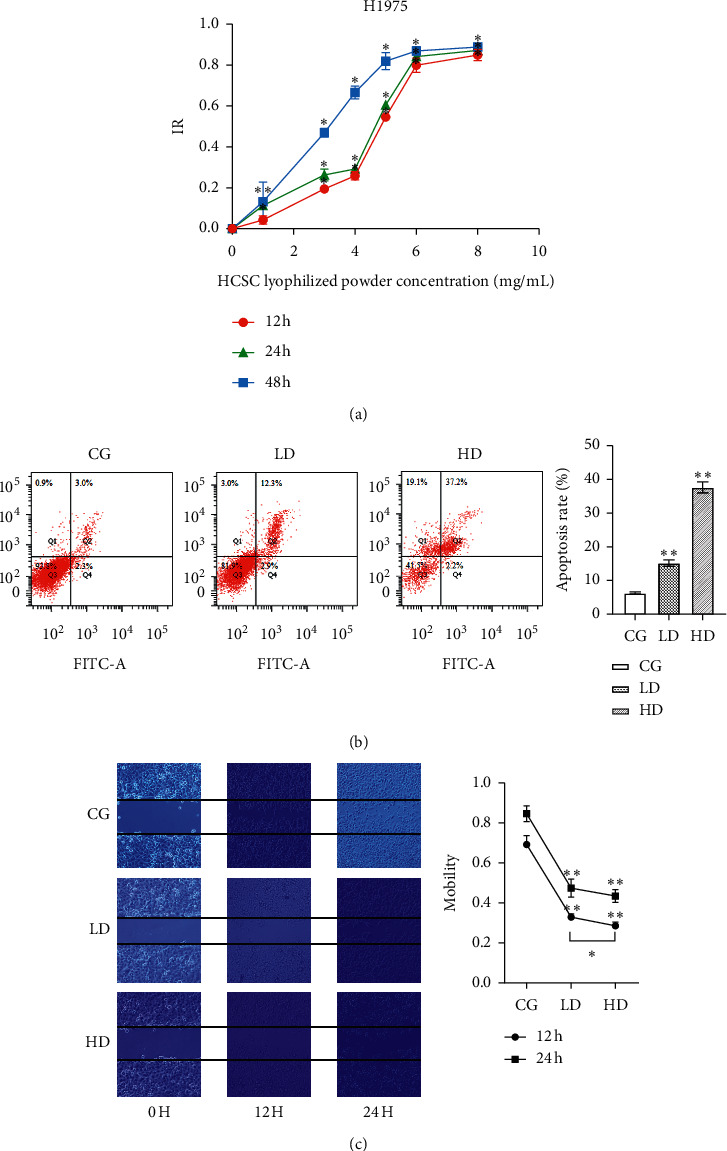
(a) Time- and dose-dependent effects of the freeze-dried HCSC powder treatment on the IR of H1975 cells; (b) the freeze-dried HCSC powder treatment induced apoptosis of H1975 cells in a dose-dependent manner; (c) time- and dose-dependent effects of the freeze-dried HCSC powder treatment on the migratory activities of H1975 cells. ^*∗*^*P* < 0.05, ^*∗∗*^*P* < 0.01 vs. CG.

The H1975 cells were treated with different concentrations of HCSC (8, 6, 5, 4, 3, and 1 mg/mL) for 24, 48, and 72 h. HCSC can inhibit cell proliferation and exhibit a dose-time effect relationship. Compared with that in the control group, in the test group, the inhibition rate increased, and the increase was statistically significant. The IC_50_ value was 5.291 mg/mL, and the IC_20_ value was 2.312 mg/mL after 24 h (Figure 7(a), 7).

#### 3.5.1. HCSC Promotes *In Vitro* H1975 Cell Apoptosis

The H1975 cells were treated with varying concentrations of HCSC (2.312–5.291 mg/mL) for 24 h. Compared with the control group, the rate of apoptosis in the test group increased significantly as the dose increased (15.17 ± 0.95 and 37.6 ± 1.64 vs. 6.33 ± 0.32) (*P* < 0.01). Thus, HCSC can indeed induce apoptosis in H1975 cells, and apoptosis may be the main cause of death (Figure 7(b)).

#### 3.5.2. HCSC Suppresses *In Vitro* H1975 Cell Migration

After 12 h of scratching, the H1975 cells in the control group migrated to the scratch site. The mobility of the LD and HD groups was lower than that of the control group (*P* < 0.01), and the mobility of the HD group decreased compared with that of the LD group (*P* < 0.05). After 24 h of scratching, the number of cells at the scratch site of the control group increased, and the scratch healing was obvious. The mobility of the LD and HD groups was reduced compared with that of the control group (*P* < 0.01); there was no significant difference in the mobility of the HD group compared with that of the LD group (Figure 7(c)).

### 3.6. Protein Expression in H1975 Cells

**Figure 8:**
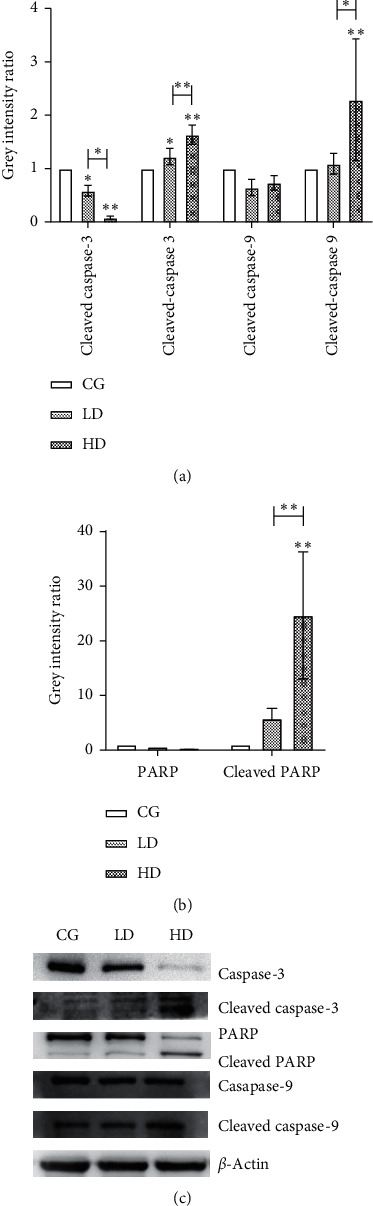
Expression levels of related proteins in H1975 cell. ^*∗*^*P* < 0.05, ^*∗∗*^*P* < 0.01 vs. CG.

Western blotting was used to detect the related proteins in the KEGG enrichment pathway, such as *β*-actin (Dawen Biotec, DW9562), caspase-3, caspase-9, and PARP (Cell Signaling Technologies, 9662S, 9508S, 9542S). We found that compared with that in the control group, in the test groups, the expression level of caspase-3 in H1975 cells was significantly decreased (*P* < 0.05), whereas the expression level of cleaved caspase-3 was significantly increased (*P* < 0.05). Compared with that in the control group, in the test groups, the expression level of caspase-3 in H1975 cells in the HD group was significantly decreased (*P* < 0.01), whereas the expression levels of cleaved caspase-3, cleaved caspase-9, and cleaved PARP were significantly increased (*P* < 0.01). Compared with that in the LD group, in the test groups, the expression level of caspase-3 in the H1975 cells in the HD group was significantly decreased (*P* < 0.05), whereas the expression levels of cleaved caspase-3 and cleaved PARP were significantly increased (*P* < 0.01) and that of cleaved caspase-9 was significantly increased (*P* < 0.05) (Figure 8).

### 3.7. Verification of Bioactive Components

**Figure 9:**
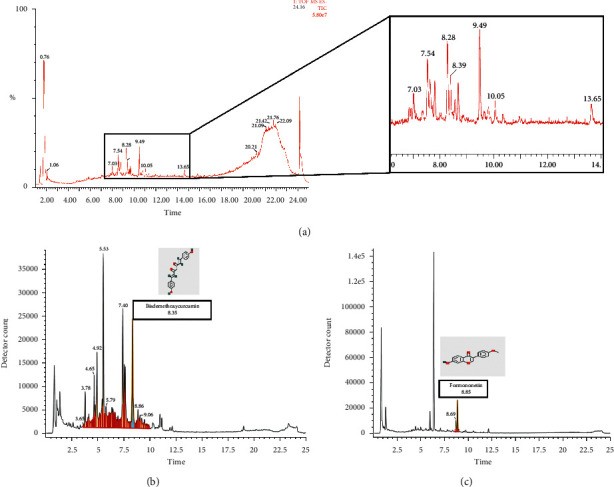
(a) The total ion current diagram of the freeze-dried HCSC powder. (b) The ion current diagram of bisdemethoxycurcumin in the freeze-dried HCSC powder. (c) The ion current diagram of formononetin in the freeze-dried HCSC powder.

The total ion current diagram of the freeze-dried HCSC powder was obtained by LC-MS (Figure 9). Upon comparison with the database, a total of 16 chemical components were identified, including terpene lactones, flavonoids and their glycosides, organic acids, and flavanols (Table 4). It covers the components in the total ion current map, which has a significantly higher response than the matrix effect. Among them, formononetin and bisdemethoxycurcumin were consistent with the potential active components retrieved from the TCMSP database.

### 3.8. Symptoms and Diseases Associated with HCSC

SymMap provides herb-symptom relationships and symptom mechanism mapping. The results are as follows: HCSC contains 15 TCM symptoms, 11 TCM syndromes, 43 modern medicine symptoms, and 150 diseases. The TCM symptoms mainly include thoracic obstruction, Zhengjia, cardiodynia, abdominal mass, and amenorrhea, whereas the TCM syndromes involve phlegm-accumulation stasis, lung-spleen qi deficiency, and qi stagnation due to liver depression. The diseases mainly include SCLC, bladder cancer, T-cell immunodeficiency, pancreatic cancer, and other cancers, and the modern medicine symptoms include chest pain, dyspepsia, neoplastic growth, angina pectoris, and menopause.

## 4. Discussion

TCM is usually applied in combination; it is a therapeutic treatment mode comprising multiple pharmacological bioactive components that cooperate with different pathways. The mechanism of HCSC with multiple components, multiple targets, and multiple pathways has limited in-depth exploration. However, the emergence of network pharmacology has solved this problem.

In this study, we collected seven potential bioactive components of HCSC, among which hederagenin is a common component of Sparganii Rhizoma and Curcumae Rhizoma. Moreover, sitosterol [29], formononetin [30], and bisdemethoxycurcumin [31] have been reported to promote apoptosis in various human lung cancer cells. Despite the general disadvantage of lower efficacy, the bioactive components of HCSC have lower toxicity and lesser side-effects than other antitumor drugs [32]. By analysing the core targets of HCSC and its enrichment analysis with neighboring genes, we concluded that it is mainly involved in the mitochondrial-mediated caspase-dependent apoptosis signaling pathway. Apoptosis is a type of programmed cell death, including the endogenous mitochondrial apoptosis pathway, exogenous death receptor signaling pathway, and endoplasmic reticulum pathway [33]. The endogenous mitochondrial apoptosis pathway is divided into caspase-dependent and caspase-independent pathways. Caspase is a family of proteolytic enzymes: caspase-9 is the initiator of endogenous apoptosis, whereas caspase-3 is the effector [34]. PARP-1 can be cleaved by caspase-3, which contributes to apoptosis [35]. GO enrichment analysis revealed that the bioactive components of HCSC may participate in apoptosis mainly by regulating the cell cycle. According to KEGG enrichment analysis, it may play an important role in treating lung cancer by regulating the P53 signaling pathway and promoting apoptosis. The results of molecular docking showed that the bioactive components in HCSC had a good affinity for the target proteins (ALB, BCL2L1, ESR1, HRAS, MAP2K1, MAPK14, and SIRT1). The docking positions were similar to the binding positions of the original ligands, which may play a role similar to that of the original ligands. Among these, sitosterol, formononetin, and bisdemethoxycurcumin, which have been confirmed to promote apoptosis, are the most effective components in combination with the target proteins. It is speculated that they are the core bioactive components of HCSC that promote lung cancer cell apoptosis.

*In vitro* experiments in H1975 cells were used to clarify the activity of HCSC in treating lung cancer and to further verify the relevant molecular mechanisms predicted by network pharmacology. Experimental results showed that HCSC can significantly inhibit the proliferation of H1975 cells, promote apoptosis, and reduce cell migration. HCSC plays an all-around role in the treatment of lung cancer and is expected to overcome the resistance of EGFR-TKI caused by the T790M mutation. Western blotting results showed that the protein expression levels of cleaved caspase-3, cleaved caspase-9, and cleaved PARP in H1975 cells after HCSC treatment were significantly higher than the levels of those in the control group. This suggests that HCSC can upregulate the mitochondrial-mediated caspase-dependent apoptosis signaling pathway by inducing the cleavage of caspase-3, caspase-9, and PARP, consistent with the aforementioned network pharmacology prediction pathway. The activity of HCSC in the treatment of lung cancer is dose- and time-dependent to a certain extent. Because there were fewer concentration gradients in our experiment, more groups still need to be set to verify the above conclusions. LC-MS confirmed that formononetin and bisdemethoxycurcumin were present in the freeze-dried powder of HCSC, which matched the prediction in the database. This suggests that the *in vitro* antitumor effect of the freeze-dried powder of HCSC may be dominated by formononetin and bisdemethoxycurcumin.

This study also explains the relationship between disease-syndrome-herb through the analysis of the SymMap database. According to the basic theory of TCM, lung cancer was historically classified into the Zhengjia category. At present, there is no unified standard for the classification of TCM syndromes in lung cancer. The deficiency of lung-spleen qi and the stagnation of qi and blood stasis are common TCM syndromes in patients with advanced lung cancer, and these syndromes often manifest as dyspepsia and chest pain [36, 37]. This proves that the TCM symptoms, TCM syndromes, modern medicine symptoms, and diseases in the results correspond with each other. It can be inferred that HCSC effectively alleviates the abovementioned TCM syndromes. Clinical studies have shown that HCSC combined with chemotherapy in the treatment of lung cancer patients with qi deficiency and blood stasis syndrome is beneficial for alleviating TCM syndromes, reducing the toxicity and side-effects of chemotherapy, and controlling tumor growth [38, 39]. However, the efficacy of HCSC on different subtypes of lung cancer and TCM syndromes requires further verification. Research on the corresponding relationship between disease, syndrome, and herbs may help explain the main TCM pathogenesis of diseases and lay the foundation for further study on the underlying mechanisms of personalised TCM treatment.

## 5. Conclusions

This research innovatively used network pharmacology and *in vitro* experiments to reach the following conclusions: (1) the core bioactive components of HCSC were sitosterol, formononetin, and bisdemethoxycurcumin; (2) HCSC can upregulate the mitochondrial-mediated caspase-dependent apoptosis signaling pathway by inducing the cleavage of caspase-3, caspase-9, and PARP; (3) the qi deficiency and blood stasis syndrome would be the most suitable for treatment with the HCSC. Thus, this study enhanced the therapeutic potential of HCSC in lung cancer.

## Figures and Tables

**Table 1:** OB and DL value of with bioactive components.

MOL ID	Bioactive components	OB	DL	Related targets	Source
MOL001297	*trans*-Gondoic acid	30.7	0.2	18	Sparganii rhizoma
MOL000296	Hederagenin	36.91	0.75	34	Sparganii Rhizoma, Curcumae rhizoma
MOL000358	Beta-Sitosterol	36.91	0.75	48	Sparganii rhizoma
MOL000392	Formononetin	69.67	0.21	49	Sparganii rhizoma
MOL000449	Stigmasterol	43.83	0.76	41	Sparganii rhizoma
MOL000906	Wenjine	47.93	0.27	10	Curcumae rhizoma
MOL000940	Bisdemethoxycurcumin	77.38	0.26	10	Curcumae rhizoma

**Table 2:** Information of core targets from bioactive components in HCSC.

UniprotID	Gene targets	Protein targets	MCODE_Score	degree
P02768	ALB	Serum albumin	10.50980392	17
P10275	AR	Androgen receptor	10.3956044	14
Q07817	BCL2L1	Bcl-2-like protein 1	10.50980392	16
P42574	CASP3	Caspase-3	10.50980392	17
Q14790	CASP8	Caspase-8	10.16190476	13
P55211	CASP9	Caspase-9	9.625	15
P24941	CDK2	Cyclin-dependent kinase 2	10.27472527	12
P03372	ESR1	Estrogen receptor	10.50980392	17
P49841	GSK3B	Glycogen synthase kinase-3 beta	11	12
P01112	HRAS	GTPase HRas	10.50980392	16
P07900	HSP90AA1	Heat shock protein HSP 90-alpha	11.42857143	17
P05412	JUN	Transcription factor AP-1	10.50980392	17
Q02750	MAP2K1	Dual specificity mitogen-activated protein kinase kinase 1	9.346405229	14
Q16539	MAPK14	Mitogen-activated protein kinase 14	9.202614379	15
P29474	NOS3	Nitric oxide synthase	10	10
P06401	PGR	Progesterone receptor	10	11
P35354	PTGS2	Prostaglandin G/H synthase 2	11.42857143	16
Q96EB6	SIRT1	NAD-dependent protein deacetylase sirtuin-1	9.625	15

**Table 3:** Docking results of bioactive components with target proteins.

Target proteins	PDB-ID	RMSD (nm)	△G (kcal/mol)
ALB	6HSC	0.9792	−10.6
BCL2L1	6RNU	1.222	−7.5
ESR1	6V87	1.1364	−9.5
HRAS	6E6C	1.2891	−9.4
MAP2K1	6PP9	1.4392	−8.1
MAPK14	6OHD	0.9642	−11.5
PTGS2	5KIR	1.4069	−9.3
SIRT1	4ZZI	1.3172	−9.7

**Table 4:** Identification of chemical components in the freeze-dried powder of HCSC by LC-MS.

Compound name	Chemical formula	Observation retention time (min)	Detector count	Response	Adduct	Observed m/z	Measured molecular mass (Da)	Molecular mass (Da)
(6S)-2-Methyl-6-[(1R, 5S)-(4-methene-5-hydroxyl-2-cyclohexen)-2-hepten-4-one]	C15H22O2	15.28	9373	5998	−H	233.1543	234.1616	234.162
Sitogluside	C35H60O6	22.57	56579	14048	+HCOO,			
+Cl	621.4356	576.4374	576.439					
Valeriotetrate A	C37H58O15	6.89	326613	49743	−H	741.3715	742.3788	742.3776
Succinic acid	C4H6O4	1.23	61216	53369	−H	117.0193	118.0266	118.0266
Benzoic acid	C7H6O2	5.23	41341	38384	−H	121.0293	122.0366	122.0368
Curcumin	C21H20O6	7.33	9845	2416	−H	367.1178	368.1251	368.126
Formononetin	C16H12O4	8.85	227089	4392	−H	267.0655	268.0727	268.0736
Prunetin	C16H12O5	9.61	60909	48019	−H	283.0615	284.0688	284.0685
Anthraquinone-2-carboxylic acid	C15H8O4	10.31	65817	54668	+HCOO	297.0408	252.0426	252.0423
Kaempferol	C15H10O6	7.69	9773	8460	−H	285.0404	286.0477	286.0477
Bisdemethoxycurcumin	C19H16O4	8.35	7087	4832	+HCOO,			
+Cl	353.1046	308.1064	308.1049					
(2E)-3,7-Dimethylocta-2,6-dien-1-ol	C10H18O	9.11	4098	3190	+HCOO	199.1335	154.1353	154.1358
3-Acetyl-6-methylpyran-2,4-dione	C8H8O4	4.87	171174	152811	−H	167.0352	168.0425	168.0423
Bisabolone-9-one	C15H22O2	9.37	3018	2568	+HCOO	279.1613	234.1631	234.162
BDBM50255128	C31H50O10	8.25	40387	2385	+Cl	617.3092	582.3398	582.3404

## Data Availability

The data used to support the findings of this study are included in the article.
